# DEER Data Analysis Software: A Comparative Guide

**DOI:** 10.3389/fmolb.2022.915167

**Published:** 2022-06-01

**Authors:** Hannah Russell, Robyn Cura, Janet E. Lovett

**Affiliations:** SUPA School of Physics and Astronomy and BSRC, University of St Andrews, St Andrews, United Kingdom

**Keywords:** EPR/ESR, DEER/PELDOR, pulsed dipolar spectroscopy, DeerAnalysis, DEERNet, Tikhonov regularization, neural networks, Gaussian models

## Abstract

Pulsed dipolar electron paramagnetic resonance (PDEPR) spectroscopy experiments measure the dipolar coupling, and therefore nanometer-scale distances and distance distributions, between paramagnetic centers. Of the family of PDEPR experiments, the most commonly used pulsed sequence is four-pulse double electron resonance (DEER, also known as PELDOR). There are several ways to analyze DEER data to extract distance distributions, and this may appear overwhelming at first. This work compares and reviews six of the packages, and a brief getting started guide for each is provided.

## 1 Introduction

Pulsed dipolar electron paramagnetic resonance (PDEPR) spectroscopy is a set of experiments that can be used to determine nanometer scale distances between paramagnetic centers by measuring their magnetic dipole-dipole interaction (Jeschke, 2012; Goldfarb and Stoll, 2018; Abdullin and Schiemann, 2020). This is a valuable technique in the study of biomacromolecules, as it can be used to ascertain their complexes and conformations. The most widely used PDEPR technique is double electron resonance (DEER, or PELDOR), (Milov et al., 1981; Milov et al., 1984; Martin et al., 1998; Schiemann et al., 2021). Applications of DEER in structural biology rely on pairwise coupling of spin-half centres with reasonably well-defined distance distributions, especially between nitroxide spin labels and this is the situation that will be discussed in this work (Haugland et al., 2017; Schiemann et al., 2021).

The DEER technique relies on the application of two microwave frequencies. The most commonly applied version of the experiment is the four-pulse (Martin et al., 1998). For four-pulse DEER, a three-pulse refocused echo sequence is applied at one frequency of the EPR absorption profile of the sample. The second frequency is applied elsewhere in the EPR spectrum with a non-overlapping, or only weakly overlapping, excitation band and is used to pump the spins. If the spins are coupled through the dipole-dipole interaction, then the pumped spins will affect the magnetic field experienced by the observed spins. The observer echo sequence has fixed time delays and the pump pulse is applied between the second and third pulses with a sequential increase in delay time. This means that the detected refocused echo over the course of the experiment, often called the DEER time trace, is modulated with the dipole-dipole coupling frequency.

Analysis of the time trace, assuming weak coupling so that the dipolar frequency is dependent on the inverse cube of the separation between centers, provides information on the distance between the coupled spins. The size of the DEER signal, the modulation depth, can provide information on the number of coupled spins (Bode et al., 2007). In theory the distances between pairwise interactions can be found from analysis of the Fourier transform of the DEER time trace, but in practice this is inaccurate, especially if there is a distribution of distances between the centers. There are a range of free-to-download software packages for providing the distance distributions and the mathematics of these fall broadly into one or more of Tikhonov regularisation, model based approaches, or trained deep-neural networks (Jeschke et al., 2006; Brandon et al., 2012; Fábregas Ibáñez et al., 2020; Keeley et al., 2022).

One major source of complication in analyzing DEER data is that the time trace is actually a convolution of the pairwise interactions, often called the form factor or, alternatively, the *intra*molecular contribution, and the interactions of randomly distributed spins within the bulk of the sample, often called the background signal, or *inter*molecular contribution. This manifests as an overall signal decay. For homogeneously distributed molecules the form is a simple exponential decay. This is the 3D background function. The background may be better described by a stretched exponential depending on the exact nature of the sample and measuring conditions. The time trace will also have a random noise associated with the measurement and may contain artefacts. One common artefact is signal associated with the so-called “2 + 1” effect, caused by the overlap of the observer and pump pulses, which appears as a distortion at the end of the time trace data set, though may be reduced if Gaussian-shaped pulses are used rather than rectangular (Teucher and Bordignon, 2018).

The analysis of the DEER signal for distances must therefore account for the pairwise and the background signals, provide error estimation, and either allow truncation of the data to remove avoidable artefacts, or not be badly affected by their presence. Additionally, the software should be intuitive to use and produce user-independent output. It may be an advantage if the package can be a stand alone executable rather than run using expensive environments, though this should be a minor consideration over the scientific aspects of providing stable and reproducible results with useful error estimation.

The recent DEER-community led white paper recommended using a combination of analysis approaches to be sure of results and included the implementation of ComparativeDeerAnalyzer (Schiemann et al., 2021). This recommendation forms the motivation behind the work presented here. We hope to answer the following questions: what are the different analysis approaches from a non-expert user perspective; how can they be used; are they reliable?

Six programs will be discussed and compared for analysis of some typical data sets. The first software used in this work is DeerAnalysis, which to date has been the most commonly used software package for analyzing DEER data (Jeschke et al., 2006) and can be downloaded from (Jeschke, 2022). This program is run through the Matlab environment and works via a graphical user interface (GUI). DeerAnalysis utilises an approximate Pake transformation (APT), Tikhonov regularization and neural network analysis via DEERNet (it no longer includes model-based approaches) (Jeschke et al., 2006; Worswick et al., 2018). In this work DeerAnalysis will be used in user-defined mode with Tikhonov regularization and using “automation” which is referred to as ComparativeDeerAnalyzer (see below). DeerLab also uses Tikhonov regularization but assumes full control over fitting the background within a given parametric model (Fábregas Ibáñez et al., 2020; Fábregas-Ibáñez et al., 2022). DeerLab is a Python-based program, and installation instructions are available at (Fábregas Ibáñez et al., 2021).

LongDistances uses a GUI and is a Windows executable (Altenbach, 2021a). It offers a number of analysis approaches including Tikhonov regularization and Gaussian fitting in a single package, but here we will focus on LongDistances’ “Model Free” method. It is available at (Altenbach, 2021b). DD also uses a GUI, but is Matlab based. It assumes that distributions are Gaussian or can be expanded into a series of Gaussian distributions (Stein et al., 2015). DD can be downloaded from (Hustedt, 2018). DEERNet uses deep neural networks to extract distance distributions in a single step, without the need for background fitting (Worswick et al., 2018). The latest version is available in Spinach (Hogben et al., 2011) [available from (Kuprov, 2021)] and also integrated as an option into DeerAnalysis (Jeschke et al., 2006). Both packages are Matlab-based. ComparativeDeerAnalyzer runs through a Windows-based executable and through DeerAnalysis using the “automation” option (which will be used in this work) (Schiemann et al., 2021). It combines results from DEERNet and DeerLab to give a mean distance distribution and a combined uncertainty. Though it should be noted that the versions of DEERNet and DeerLab used by ComparativeDeerAnalyzer are often not the most up-to-date or reliable versions available.

Attempting to learn to use these various software packages and extract results for onward presentations may appear a daunting prospect to the uninitiated user. This paper has the following layout: first, we hope to lay the groundwork to simply import and analyze a DEER data set, and also give insight into the output files to clarify their usability for future plotting and comparison for each software; then, the methods carried out for analysis comparisons are presented, followed by the results themselves; finally, there will be a discussion regarding the results, applicability and usability of each analysis method.

## 2 Materials and Methods

### 2.1 Getting Started

#### 2.1.1 DeerAnalysis

DeerAnalysis (Jeschke et al., 2006) is a Matlab-based package that exists in two modes; “automation” (ComparativeDeerAnalyzer) and user-defined. It is available for download from (Jeschke, 2022), and an accompanying manual can be found within the download folder. Here we introduce user-defined mode but using as many program-calculated values as possible. To begin, obtain the program from the web and set it in the Matlab path, then type DeerAnalysis in the command window to open the GUI. The version of DeerAnalysis discussed here is DeerAnalysis2022 which has significant differences to earlier versions, as detailed in the downloadable documentation.

**FIGURE 1:**
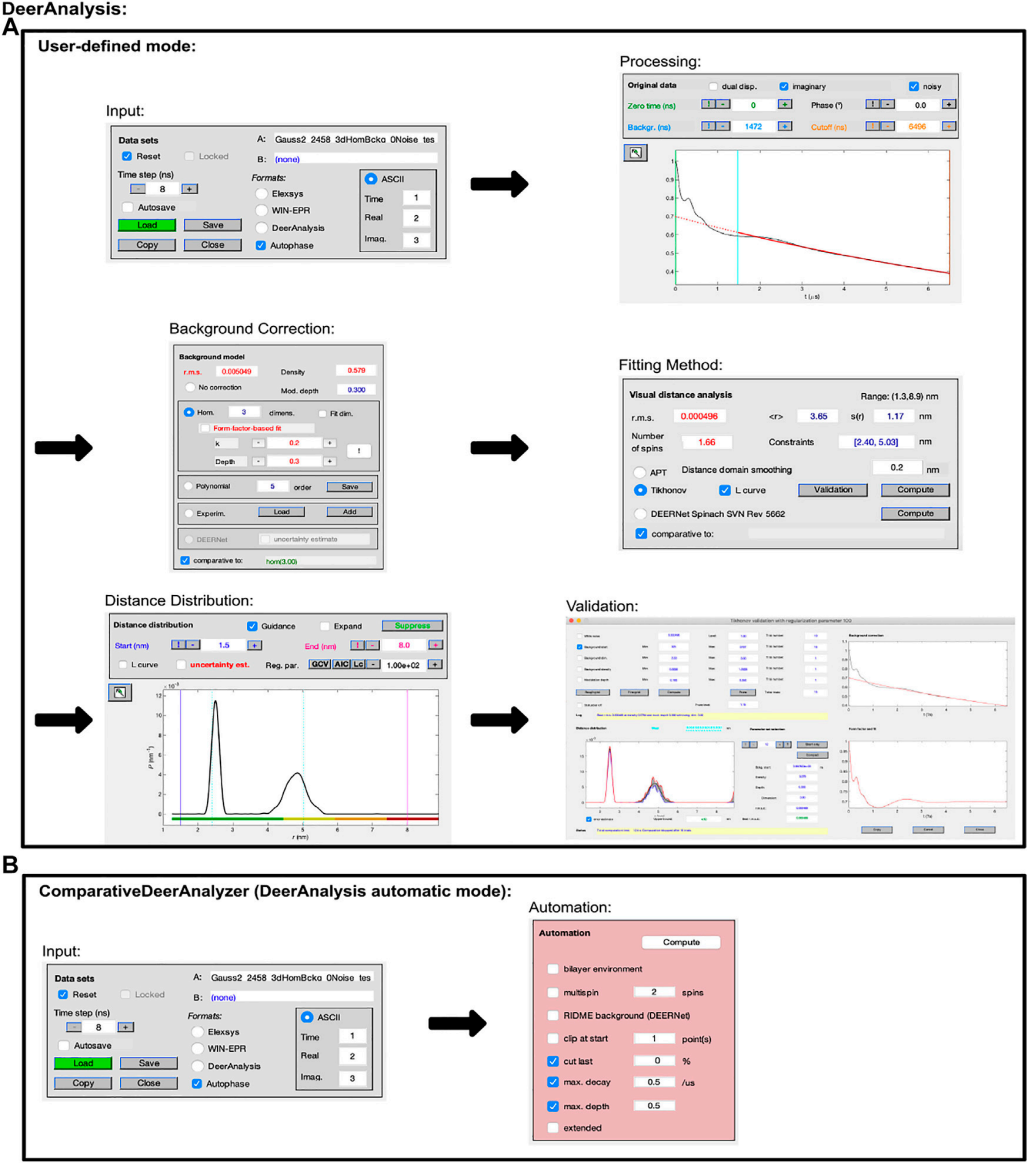
DeerAnalysis2022 workflow. The program is Matlab-based and available on all operating systems. Data set shown is simulated noiseless, which was an ASCII file. For users using Elexsys file types, “Elexsys” should be selected in place of “ASCII”; **(A)** “user-defined” mode; **(B)** “automation” mode (otherwise known as ComparativeDeerAnalyzer, which is also available as a standalone Windows executable).

The following paragraphs describe the workflow shown by Figure 1A. The methodology for user-defined mode is as follows; the user uploads a measured data set; the program accepts input data files in the form of Bruker Elexsys (.dta/.dsc), WIN-EPR, and ASCII. DeerAnalysis will automatically select a zero time and phase correction value. The criteria for these are described in the DeerAnalysis documentation. That being said, any value between 0 and the entire length of data can be input to the text box and selected as the zero point. Likewise, any value can be selected as the phase correction, background start, and amount of data cut from the end. DeerAnalysis’s user-defined mode includes the option of automatically determining the “optimal” value for each of these fitting parameters by pressing the “!” button present next to each. After selecting parameters, a background should be fitted. The default background is a 3D homogeneous model but other options are available, including an experimentally determined one.

At this stage the distance distribution box contains the result from the approximate Pake transformation (APT) which is fast to calculate but not very accurate (Jeschke et al., 2002). DeerAnalysis offers the user several different approaches to analyze the data, and this is selected using the panels. Probably the most common approach to analyzing DEER time traces with DeerAnalysis is to use Tikhonov regularization with the L-curve method for determining the regularization parameter (Chiang et al., 2005; Edwards and Stoll, 2018). The distance distribution graph also contains colored markers along the *x*-axis which represent a measure of the confidence in the distance presented based on the length of the time trace. This is described further in the accompanying manual.

DeerAnalysis uncertainties when Tikhonov regularization is used are calculated via “Validation”. Selecting this opens a new GUI and the parameters to vary are clearly set out, for example, background start value, white noise level etc. The program then runs through the calculation for each of the varied parameters and this gives a measure of uncertainty.

The figures can be saved directly, or data files of the results are saved as six text files, further described in the documentation, by pressing the “save” button. However, after validation, the best fit determined by the optimal parameters determined previously will be overwritten in favor of the validation fit. We therefore recommend saving both sets of results separately and that your results plot contains the best fit and the validation uncertainties. The final output data can then be plotted again using the Matlab *plot* function.

#### 2.1.2 DeerLab

DeerLab is a script-based analysis package run through the Python environment, and accepts Bruker Elexsys (.dta/.dsc) and ASCII format input files. The DeerLab documentation (Fábregas Ibáñez et al., 2021) provides clear and straightforward instructions for the installation and general use of the program. The version of DeerLab described here is pre-release 0.14.0, meaning that it is not the final stable version of the program and is still under development.

**FIGURE 2:**
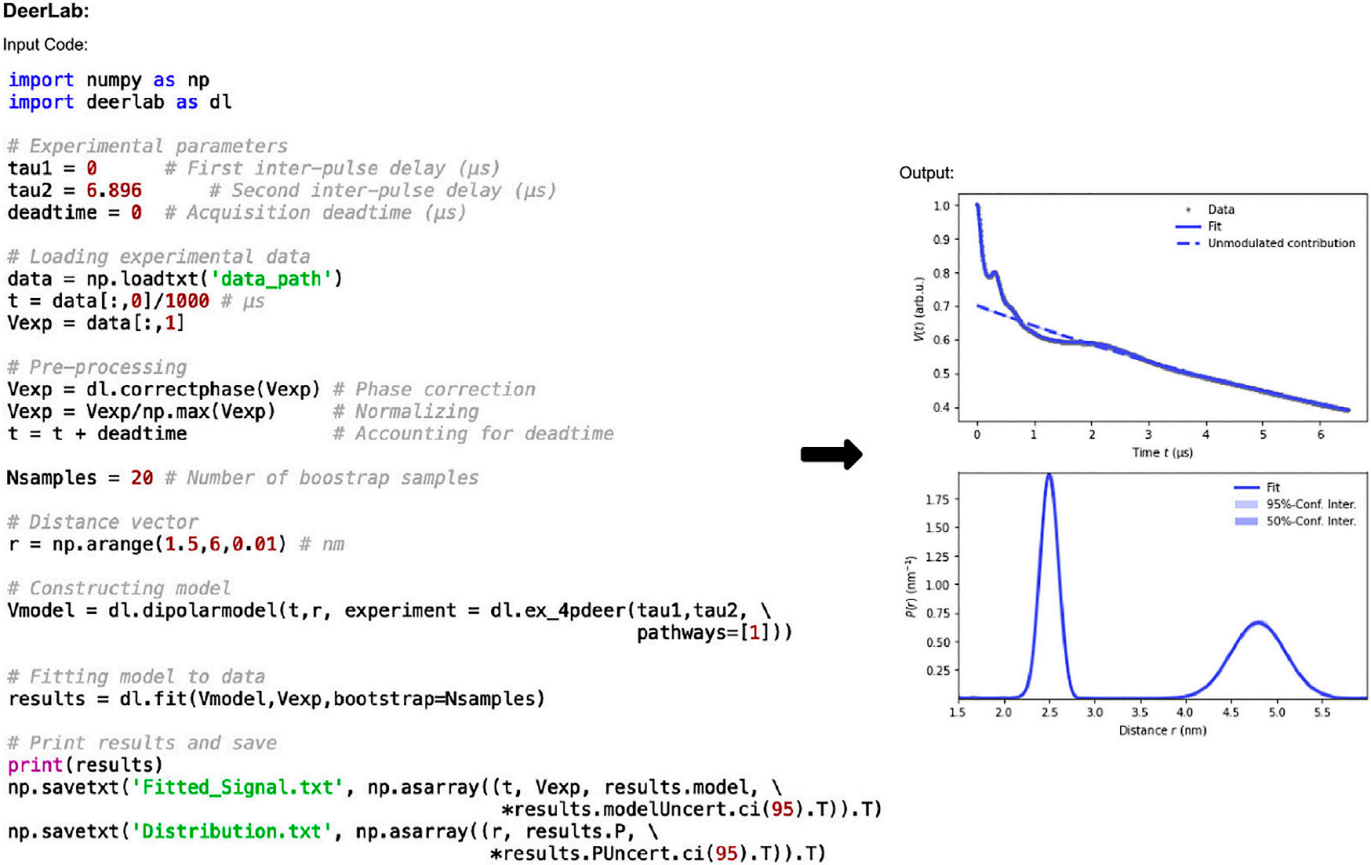
DeerLab pre-release v0.14.0 workflow. The package is Python-based and available on all operating systems. Data set shown is simulated noiseless, which was an ASCII file. For users using Elexsys file types, the lines of code “data_path = ‘/…/data/datafile.DTA’” and “t,Vexp = dl.deerload (data_path)” should be used in place of “data = np.loadtxt (‘data_path’)” and the two following lines in panel one.

The code in Figure 2 shows how data is imported and processed. In this case the code implements a 3D background and Tikhonov regularization. It can be adapted for numerous experiments, fitting methods, and models. The functions used in Figure 2 are: *correctphase* which minimizes the imaginary and maximizes the real components of the signal; *dipolarmodel* which requires the user to input time data (*t*), which should account for the deadtime, alongside the distance range, and an experiment model. The distance range, commonly defined as *r*, is set by the user and should be determined such that the lower limit is dependent on the pulse bandwidth and time increment. In this example the lower limit is 1.5 nm which is typical for a 12 ns rectangular pump pulse. The upper limit should consider the overall time trace length and the expected distances present in the sample, here it is set at 6 nm. A range of experimental models are available for use. In this work “ex_4pdeer” is used which assumes a 4-pulse DEER experiment. The parameters required by this function are the *τ*_1_ and *τ*_2_ time delays of the experiment and the number of dipolar pathways, which will be based on the experiment and the type of pulses used. For 4-pulse DEER, a single pathway will be required for data without a 2 + 1 effect component, but if this artefact is present, then a second pathway should be included by changing “pathways=[1]” to “pathways= [1,2].” Without giving the *dipolarmodel* function any additional information, it will assume a non-parametric distribution and a 3D homogeneous background, which are common analysis methods for DEER data. The non-parametric method is solved using Tikhonov regularization. Rather than using the L-curve method typically implemented in DeerAnalysis, the regularization parameter is determined according to the Akaike information criterion (AIC) (Akaike, 1974) by default. Parametric model options such as Gaussian, are also available. DeerLab uses least-squares fitting to determine the optimal parameter values to fit the data for the distance distribution, background, and experiment. Finally, the model set up using *dipolarmodel* can be fit to the phase-corrected input data using the *fit* function. While these models and functions provide accurate results, the user should always check that the parameter values and general goodness-of-fit indicators make sense: implementing “print(results)” will mean that DeerLab presents these results too.

The default method for error-analysis in DeerLab is covariance-matrix uncertainty. Given its speed and ease, the calculation is included in all DeerLab fit functions, and they return covariance-based errors for all fitted parameters. For much more accurate uncertainty estimates, however, DeerLab offers bootstrap analysis (Efron and Tibshirani, 1986). Though this is significantly more computationally expensive, it is the recommended method and is the one used in this work. Bootstrapping is easy to implement in DeerLab, and requires only “bootstrap = Nsamples” to be added as an input in the *fit* function for use.

The fitted data can be accessed using “results” where “results.model” and “results.P” return the fitted dipolar signal, and distance distribution results, respectively. To save the data to plot it elsewhere, the final two lines of code in panel one of Figure 2 can be used to save the fitted dipolar signal and distance distribution results, with associated uncertainties, respectively. The data is saved as a .txt file.

#### 2.1.3 LongDistances

LongDistances exists only as a Windows executable program. Its installation requires the user to visit the Hubbell lab website (Altenbach, 2021b) and download the zip file containing all their offered software. Upon download, LabVIEW 2020 Runtime “lite” should be installed, alongside the LongDistances program. Full installation instructions are available on the web (Altenbach, 2021c), and a further *readme* document is available in the zip file for extra installation instructions. The version of LongDistances described here is v1073.

**FIGURE 3:**
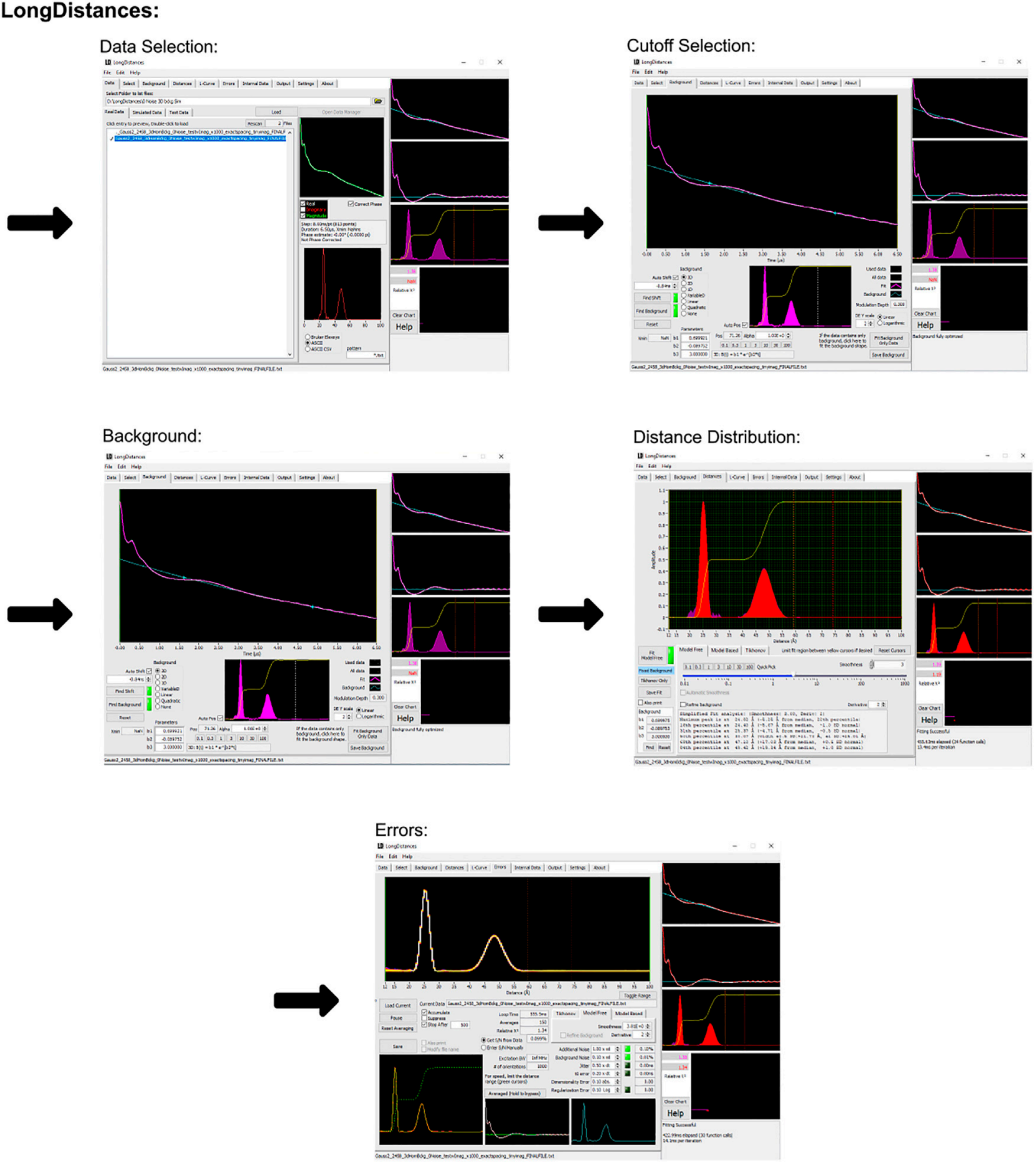
LongDistances workflow. The package was developed in LabVIEW and is available as a Windows executable only. Data set shown is simulated noiseless, which was an ASCII file. For users using Elexsys type files, “Bruker Elexsys” should be selected in panel one in place of “ASCII”.

The LongDistances GUI consists of a series of tabs leading to different stages of the analysis. A workflow of this process can be seen in Figure 3. First is the “data” tab, here, the user’s data can be selected and imported to the program. The program’s accepted input files are Bruker Elexsys (.dta/.dsc) and ASCII formats. The next tab is entitled “select” and is where the time range for analysis is selected. LongDistances implements a largely mouse-based control so that to cut data from the time trace, the cursor can simply be used to cut data from either side of the DEER signal. Next, a background should be fit to the data in the “background” tab. The zero time is automatically calculated and applied to the data, but can be overwritten by the user, and the “find background” button should be pressed to fit a background. There are a number of background model options available. After a background has been determined, the user can navigate to the “distances” tab. Here, several fitting options are available, including Tikhonov regularization and a sum of Gaussians model-based approach, but the recommended process for distance distribution determination is the model-free method, which is described by the author as being similar to non-negative Tikhonov regularization. This approach uses a “smoothness” parameter. To determine the ideal value for this parameter, the user should use their own intuition and choose a value “by-eye”. The value should be one that smooths the resulting distribution but should not be so high a number that it causes the result to become over-broadened. The smoothness parameter can be altered by the user using the slider, text box or selecting a provided value.

LongDistances offers the user the ability to calculate uncertainties on most fit parameters. Repeated analysis with varying parameters is carried out to determine the uncertainties in the data. This feature is available within the “error” tab. Finally, for future use of the results, five main outputs are provided upon saving the analysis. These are the named “filename_” followed either by DEER, DIST, DIST_ERROR, or RSLT, depending on what data the file contains, or the fifth output which is a .png.

#### 2.1.4 DD

DD is a Matlab-based analysis package specializing in Gaussian distributions (Brandon et al., 2012; Hustedt et al., 2021). The version of DD described here is 7C, which is available from (Hustedt, 2018) and includes an accompanying user manual.

**FIGURE 4:**
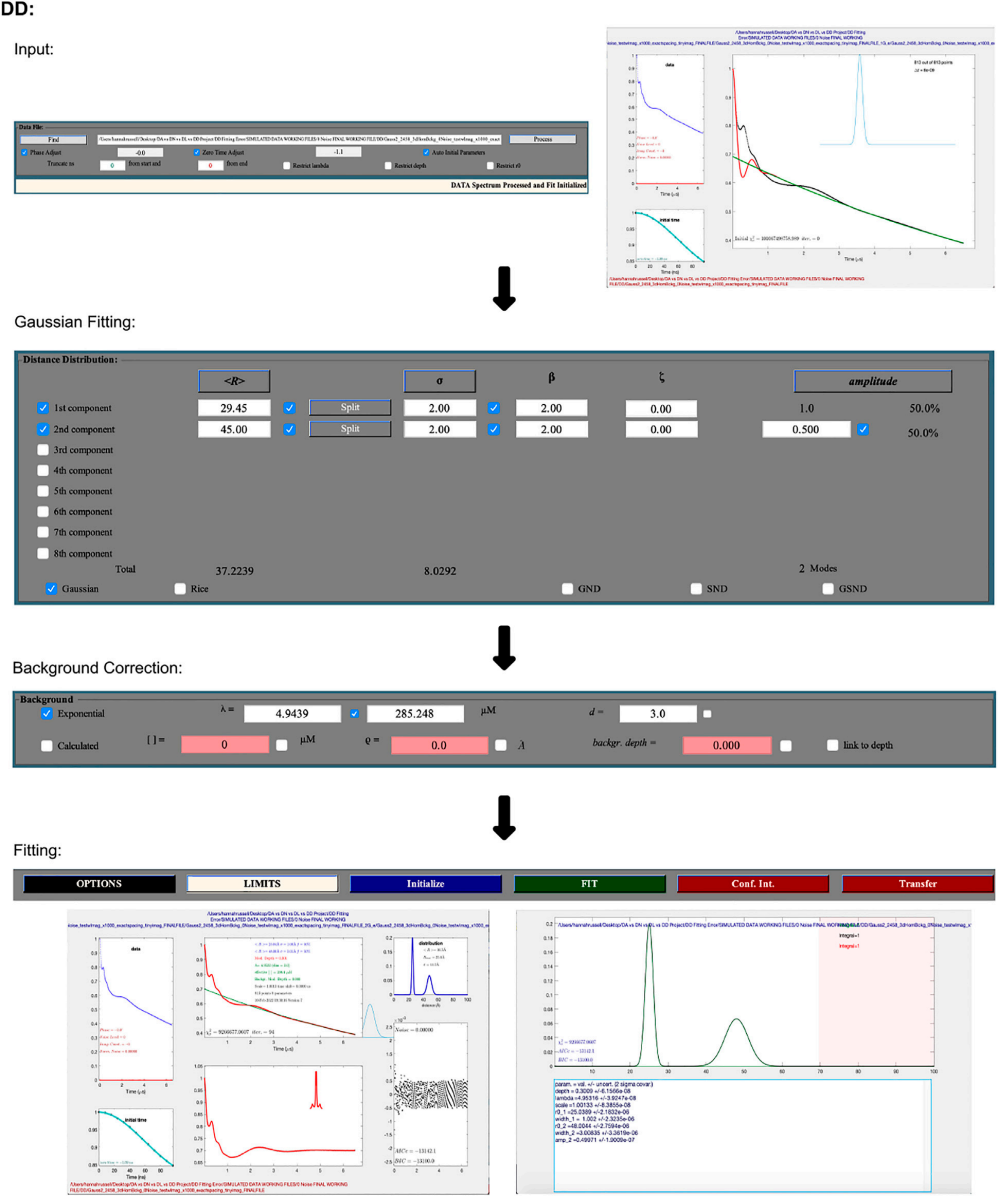
DD workflow. The package is Matlab-based and available on all operating systems. Data set shown is simulated noiseless, which was an ASCII file. No additional steps or changes are required for importing different file types.

The following procedure is illustrated as a workflow in Figure 4. DEER data is uploaded using the “Find” button in the “Data File” panel. It accepts input files in the form of Bruker Elexsys (.dta/.dsc) and ASCII. Automatically, DD will optimize the zero time and phase correction values, but a “process” option is also present that will re-calculate these values, which may be necessary. An example of an occasion where this may be necessary is post any truncation of the data, which can be achieved by typing values into the text boxes in the “Data File” panel. The user is then presented with a new pop-up window that shows the raw data and newly zero time and phase corrected result.

The second and largest box within the GUI lets the user select the number of Gaussians they want to fit the data with, while the third box fits the background. The pre-selected background model assumes a three-dimensional distribution of spins. DD also gives the user the option to fit the background with a calculated model that takes into consideration the spin concentration and the excluded volume of the sample. After selecting the fitting process and parameters, which can be edited by typing values directly into the text boxes, the data is fit by pressing the green “FIT” button.

To determine the optimal number of Gaussians to fit the distance distribution to, a number of statistical parameters should be referred to. DD offers any number of Gaussians from 1–8, and according to the DD user manual (Hustedt, 2018), the “best” fit should be determined by the chi-square, *χ* ^2^ (goodness of fit); the closer the value is to 1, the better the fit. The second criteria for determining the optimal fit is the Bayesian information criterion (BIC) (Schwarz, 1978), the lowest BIC indicates the best fit.

DD calculates errors by determining the uncertainty using the delta method, wherein the partial derivative of the best fit is taken at a particular distance with respect to all fit parameters (Hustedt et al., 2018).

To save data in DD, the user presses the “write” button. By default, data is set to save to a single Excel document with a number of sheets containing the various output data sets, but this can be changed by pressing the "options" button and navigating to the output tab where the data can be saved as either ASCII or .mat files.

#### 2.1.5 DEERNet

The version of DEERNet discussed here is the full script based version which is available through Spinach 2.6.5625 and can be downloaded from (Kuprov, 2021). Installation then only requires the user to add Spinach to their Matlab path. There exist a series of links in the downloaded folder with further details on installation and instructions on how to get started.

**FIGURE 5:**
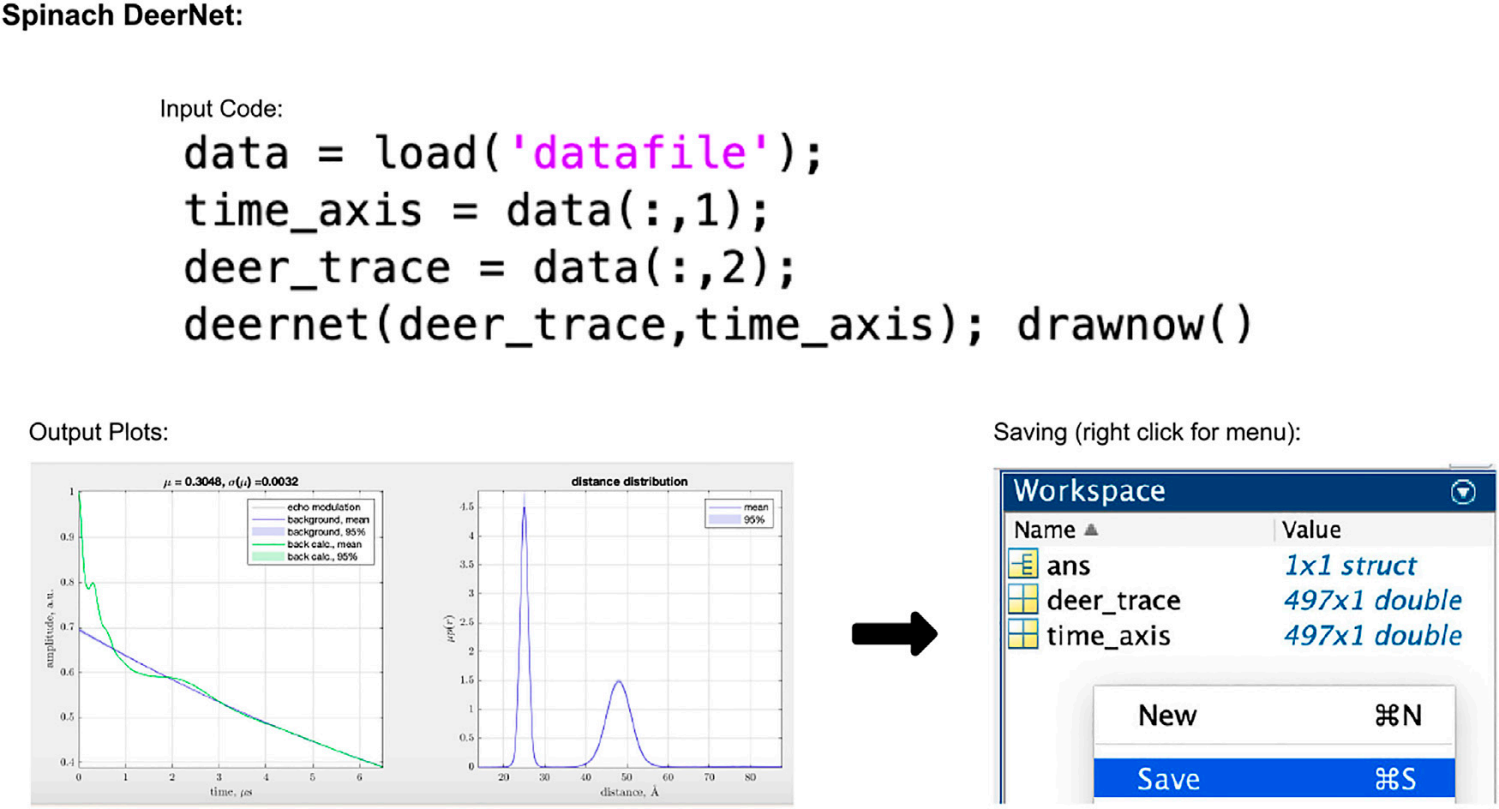
Spinach 2.6.5625 DeerNet workflow. The package is Matlab-based and available on all operating systems. Data set shown is simulated noiseless, which was an ASCII file. For users using Elexsys type files, the first three lines of the input code should be replaced with the single line “[deer_trace, time_axis] = elexsys2deernet (‘datafile’)”. Note that no file extensions should be added to the end of “datafile” when using this function. In the output plot panel, the numbers above the DEER trace signified by *μ* and *σ*(*μ*) represent the modulation depth, and the standard deviation in the modulation depth, respectively.

In the script-based version of DEERNet, the user's only input to the process is to select the data, which can be done using the code given in Figure 5. The accepted input file formats are Bruker Elexsys (.dta/.dsc) and ASCII. After running this piece of code, the user will be presented with a pop-up window showing the fitted background and extracted distance distributions both with 95% uncertainty bounds (for an example, see Figure 5). In DEERNet, distance distribution uncertainties are calculated through the training of the neural networks on different, randomly generated, data inputs and performing statistical analysis on their output results.

Data can be saved from Matlab into a .mat file which saves the input and output data. This must be further dealt with by the user if they wish to have an ASCII-type data file.

Pre-packaged DEERNet runtime libraries are used in ComparativeDeerAnalyzer and DeerAnalysis.

#### 2.1.6 ComparativeDeerAnalyzer

ComparativeDeerAnalyzer is available from (Jeschke, 2022), where the download includes a short explanatory document (Schiemann et al., 2021). It is available both as a Windows-based executable (version 2.0) and in DeerAnalysis2022 when run using the “automation” option. In this version of ComparativeDeerAnalyzer, DeerLab is run through version 0.9.1 and DEERNet through Spinach SVN Rev 5662. ComparativeDeerAnalyzer aims to provide a solution to the existing issue in the field of different results being obtained by different analysis packages. To combat this, the package offers a, so-called, consensus result which is determined as being the mean of DeerLab’s Tikhonov regularization and DEERNet acquired distributions, where the associated uncertainty is made up of both the method’s errors. The DEERNet uncertainties are calculated via training of the neural networks with simulated data sets, and the variation in their results is used to track the uncertainties, while the Tikhonov errors are determined as being twice the standard deviation of 11 different, equally spaced, backgrounds over 55 iterations. For each background, 5 trials are performed wherein the noise is varied.

The program has limited user input and this is controlled through the checkbox options that can be seen in panel two of Figure 1B for DeerAnalysis 2022. This includes the option for the user to cut-off a pre-determined amount of the data, but otherwise ComparativeDeerAnalyzer will determine whether to remove data points. We note that the degree of input has increased in the newest releases, since the original version allowed for none (Schiemann et al., 2021).

ComparativeDeerAnalyzer mode in DeerAnalysis is accessed by loading the data, as shown in the first panel of Figure 1B, and pressing “compute.” A prompt will guide the user to choose a datafile to import (Bruker Elexsys (.dta/.dsc) or ASCII accepted). All parameters and calculations are carried out directly using DeerLab 0.9.1 Tikhonov regularization and DEERNet Spinach SVN Revision 5662.

The output is a PDF containing the individual fits and distributions of the data, and also plots of the consensus result based on the two. The parameters used for the analysis are also presented at the end of the document. Also shown at the bottom of the PDF are the save locations of the output data. A number of csv files are saved: these include the distributions from DEERNet, DeerLab, and the consensus fits and distributions. Further files include the meta-data and also the results in.mat format.

### 2.2 Versions

In this work the following operating systems, coding packages, and analysis packages were used; MacOS Mojave Version 10.14.6, Windows 10 (used for LongDistances only), Matlab R2021a, Python version 3.8.5 through Spyder 5.0.5 via Anaconda Navigator 2.04, DeerAnalysis2022 (which includes ComparativeDeerAnalyzer 2.0), DEERNet via Spinach version 2.6.5625, DD version 7C, DeerLab pre-release version 0.13.2 for data simulation, DeerLab pre-release version 0.14.0 for data analysis, 64 bit LabVIEW 2020 “lite” Runtime engine, and LongDistances1073.

### 2.3 Generating Simulated DEER Data

DeerLab v0.13.2 was used to simulate DEER data containing two distinct distance distributions. Simulations were used to produce sets of data with known distance distributions to allow comparison to the results from the analysis programs. To do this, the “dd_Gauss2” function was used with parameters (2.5, 0.1, 0.5, 4.8, 0.3, 0.5) corresponding to two Gaussians (Gaussian 1: mean distance 2.5 nm, standard deviation 0.1 nm, relative weight 0.5; Gaussian 2: mean distance 4.8 nm, standard deviation Gaussian 0.3 nm, relative weight 0.5). The modulation depth was set at 0.3 and the time axis was set between 0 and 6496 ns with 8 ns resolution. Noise could be added and was found to be necessary on the imaginary part for DD to process the data. The data called noise-free in fact has a noise level of 10^−7^ on the imaginary part. Data with noise added had a 0.01 level. The background function can be varied. 3D backgrounds were created with the “dd_hom3d” function with a spin concentration of 300. 2D backgrounds were created with the “bg_homfractal” function with a pumped spin fractal concentration of 300 × 10^−7^ *μ*mol/dm^*d*^ and a fractal dimension, *d*, of 2. Data from these analyses are not shown in this paper though results are discussed briefly.

### 2.4 Experimental DEER Data

Two sets of different experimental DEER data were used for testing and demonstrating the different packages. The data were all from nitroxide Q-band 4-pulse DEER and the samples are the copper amine oxidase protein from *Arthrobacter globiformis* (AGAO) and spin-labelled DNA. Experimental details are given in the relevant publications and the data are freely available with links presented in the publications (Hardwick et al., 2020; Russell et al., 2021).

### 2.5 Data and Fitting Parameters

**FIGURE 6:**
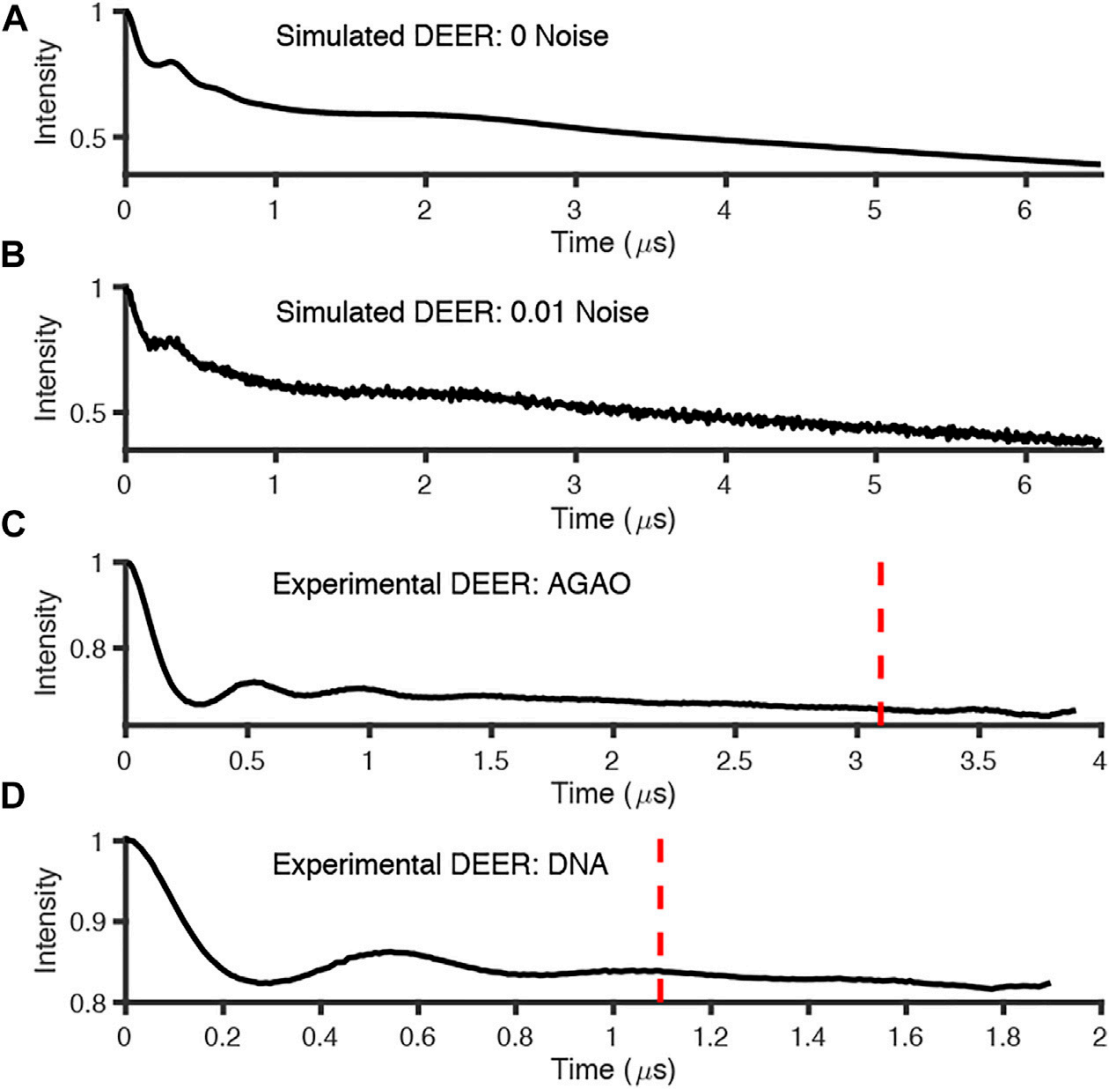
Simulated and experimental DEER traces where red dotted line signifies 800 ns cut from end points; **(A)** simulated noiseless; **(B)** simulated noisy; **(C)** experimental AGAO; **(D)** experimental DNA measurements. Further details of the data sets in the main text.

The simulated data and experimental DEER time traces (Figure 6) were analyzed using all six packages following the procedures set out in the Getting Started section. The parameters and selected output variables are presented in Tables 1–3 for DeerAnalysis, DD and LongDistances respectively. The key to the “Data set” column is that data set 1 is simulated with no noise; Data set 2 is simulated with 0.01 noise level; data set 3 is the experimental data from spin-labelled AGAO; data set 4 is the experimental spin-labelled DNA data.

**TABLE 1:** DeerAnalysis parameters.

Data set	Maximum time (ns)	Zero time (ns)	Background start (ns)	Tikhonov parameter	Modulation depth
1	6496	0	1472	100	0.300
2	6496	0	2592	1000	0.314
3	3896	331	952	7.94	0.287
	3096	331	544	25.1	0.286
4	1896	330	336	10	0.141
	1096	330	352	10	0.139

**TABLE 2:** DD parameters.

Data set	Maximum time (ns)	Zero time (ns)	No. of Gaussians	Modulation depth
1	6496	0.0	2	0.301
2	6496	0.0	2	0.307
3	3896	330.7	3	0.289
	3096	330.7	3	0.289
4	1896	330.2	3	0.142
	1096	330.2	2	0.142

**TABLE 3:** LongDistances parameters.

Data set	Maximum time (ns)	Zero time (ns)	Smoothness parameter	Modulation depth
1	6496	0.0	3	0.300
2	6496	0.0	3	0.304
3	3896	330.7	3	0.293
	3096	330.7	3	0.287
4	1896	330.1	3	0.149
	1096	330.1	1	0.147

In all the DeerLab fits, the function “ex_4pdeer” was used to assume a 4-pulse DEER experiment, and *dipolarmodel* used no input arguments so as to run with the default non-parametric (*i.e.,* Tikhonov regularization) and 3D homogeneous background fitting models. For the simulated data sets (noiseless and noisy) only a single pathway was applied, as no 2 + 1 effect artefact is present, and the experimental parameters *τ*_1_ = 0 *μ*s, *τ*_2_ = 6.896 *μ*s and deadtime = 0 *μ*s were used for both sets of simulated data. For the experimental data sets (AGAO and DNA), two fits were conducted in each program, one with 0 ns cut from the data, and one with 800 ns cut, to remove the 2 + 1 effect. In DeerLab, a second pathway can be included in the dipolar model to account for this artefact. Therefore, the 0 ns cut data used two pathways, while the 800 ns cut data used one. The AGAO experimental parameters were *τ*_1_ = 0.4 *μ*s, *τ*_2_ = 4 *μ*s and deadtime = 0.08 *μ*s and for DNA these were *τ*_1_ = 0.4 *μ*s, *τ*_2_ = 3 *μ*s and deadtime = 0.08 *μ*s. In all analyses, bootstrap analysis was used to calculate the uncertainties in the results. The DeerLab documentation encourages the use of 1000 bootstrap samples to avoid non-convergence of the confidence intervals. However, due to this being computationally expensive and requiring greater lengths of time to run, only 20 bootstrap samples were used in this work as this proved to be a sufficient number to ensure convergence of the confidence intervals for our data sets.

DEERNet requires no user input or fitting parameters. Variations of the code in Figure 5 were used, and similarly, ComparativeDeerAnalyzer was run without any user input.

## 3 Results

### 3.1 Results From Simulated DEER Data

**FIGURE 7:**
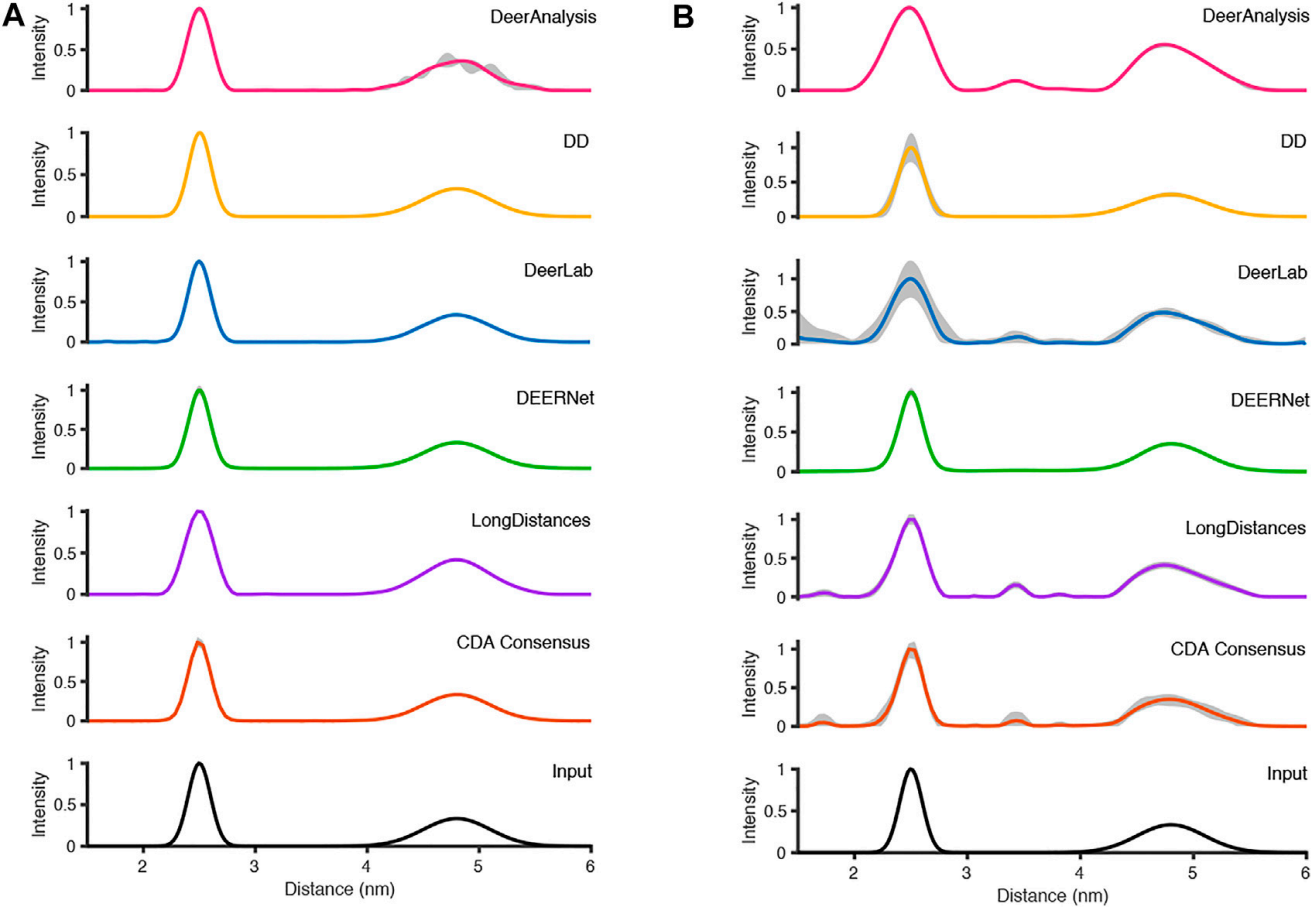
The analysis of the simulated DEER time traces using the various analysis packages (CDA Consensus is the consensus result from ComparativeDeerAnalyzer) and the original input distance distributions are shown at the bottom: **(A)** noiseless data; **(B)** noisy data.

The six packages were tested on simulated DEER time traces which had been generated from two equally weighted non-overlapping Gaussian distributions with different standard deviations. The two Gaussian’s were centered at 2.50 and 4.80 nm with respective full-width-at-half-maximum (FWHM) values of 0.24 and 0.71 nm. The overall mean of the input data was 3.65 nm. The equal weighting of the peaks means that the second, broader peak is 33% the size of the first peak. The results of processing the simulated data are presented in Figure 7 and statistics are given in Tables 4 and 5. The tables present the overall mean of the distance distributions from each of the results, and the mean and FWHM for the two most probable distance distribution peaks (which correspond to the expected input peaks in all cases). The tables also present a mean and standard deviation for results from all the packages in each of these categories.

**TABLE 4:** Results from each program for the simulated DEER-with-no-noise data set.

	Overall mean (nm)	Mean main peak 1 (nm)	FWHM main peak 1 (nm)	Mean main peak 2 (nm)	FWHM main peak 2 (nm)	Height of peak 2 compared to peak 1 (%)
DeerAnalysis	3.65	2.50	0.26	4.80	0.69	36
DD	3.65	2.50	0.24	4.80	0.71	33
DeerLab	3.64	2.50	0.24	4.80	0.70	34
DEERNet	3.69	2.50	0.23	4.80	0.70	33
LongDistances	3.65	2.50	0.30	4.80	0.70	42
CDA Consensus	3.67	2.50	0.24	4.80	0.69	34
Mean of Results	3.66	2.50	0.25	4.80	0.70	35
Standard Deviation	0.02	0.00	0.03	0.00	0.01	3.4

**TABLE 5:** Results from each program for the simulated DEER-with-noise data set.

	Overall mean (nm)	Mean main peak 1 (nm)	FWHM main peak 1 (nm)	Mean main peak 2 (nm)	FWHM main peak 2 (nm)	Height of peak 2 compared to peak 1 (%)
DeerAnalysis	3.60	2.47	0.45	4.84	0.75	56
DD	3.62	2.50	0.26	4.80	0.76	32
DeerLab	3.56	2.58	0.38	4.84	0.75	48
DEERNet	3.62	2.50	0.25	4.82	0.67	35
LongDistances	3.60	2.48	0.30	4.84	0.74	41
CDA Consensus	3.59	2.49	0.26	4.83	0.72	35
Mean of Results	3.60	2.50	0.32	4.83	0.73	41
Standard Deviation	0.02	0.04	0.08	0.02	0.03	9.2

Figure 7A shows that the output from the programs were all very similar when the input DEER time trace had effectively no noise present. The uncertainties were barely visible except for the DeerAnalysis result where the longer, broader distance had some oscillatory variation following the validation procedure. Table 4 further highlights how well the packages reproduced the input distance distributions with the only variation coming from very small differences in the FWHM values and in the relative heights of the two peaks. The first peak from the LongDistances analysis was slightly broadened and varied by more than one standard deviation from the mean (0.30 nm compared to the input value of 0.24 nm and the mean from all results value of 0.25 nm). This also led to the % height of the second peak being a little larger than the mean or input value.

The results from the different packages varied more for the simulated data with a modest level of noise, and the results are shown in Figure 7B. By visual inspection, the Gaussian fitting from DD produced the input well with some uncertainty visible on the shorter, more narrow, distance. DEERNet also gave back the input very well and with almost no uncertainty. The other packages, which used Tikhonov regularization or similar, give an output with an erroneous apparent distance centered around 3.3 nm. Though only in DeerAnalysis and LongDistances was this seen as having certainty, and therefore the results have been affected by the presence of appreciable noise on the simulated data. The DeerLab result had a much larger degree of uncertainty compared to the noiseless input data result shown in Figure 7A. The uncertainty had also increased for ComparativeDeerAnalyzer and DD but appears reduced for the DeerAnalysis result. The shape of the longer distance peak appeared visibly asymmetric for the results from DeerAnalysis, DeerLab, LongDistances and ComparativeDeerAnalyzer.

Some statistical results from each package are shown in Table 5. The standard deviations were small at less than 0.1 nm, meaning that all packages produced a result in good agreement with the rest. The overall mean of the entire distribution from each package was 3.60 nm with a standard deviation of 0.02 nm, which was shorter than the overall mean of the input (3.65 nm). The mean of the first prominent peak was 2.50 nm with a standard deviation of 0.04 nm. This fit the mean for this peak from the input data well. Only the result from DeerLab varied significantly: approximately two standard deviations away. The second prominent peak was at 4.83 nm with a standard deviation of 0.02 nm: almost all the programs slightly overestimated the mean position of the peak. The widths of the main peaks were slightly broader than the input values. The height ratio of the two peaks varied much more than for the results from the input data with no noise. In particular, the DeerAnalysis result was notably far from the input value.

### 3.2 Results From Experimental DEER Data

The experimentally measured DEER data used in these tests were measured to good signal-to-noise levels and have both visible modulations (indicative of reasonably narrow distance distributions) and visible artefacts at the end of the time traces (Figure 6).

**FIGURE 8:**
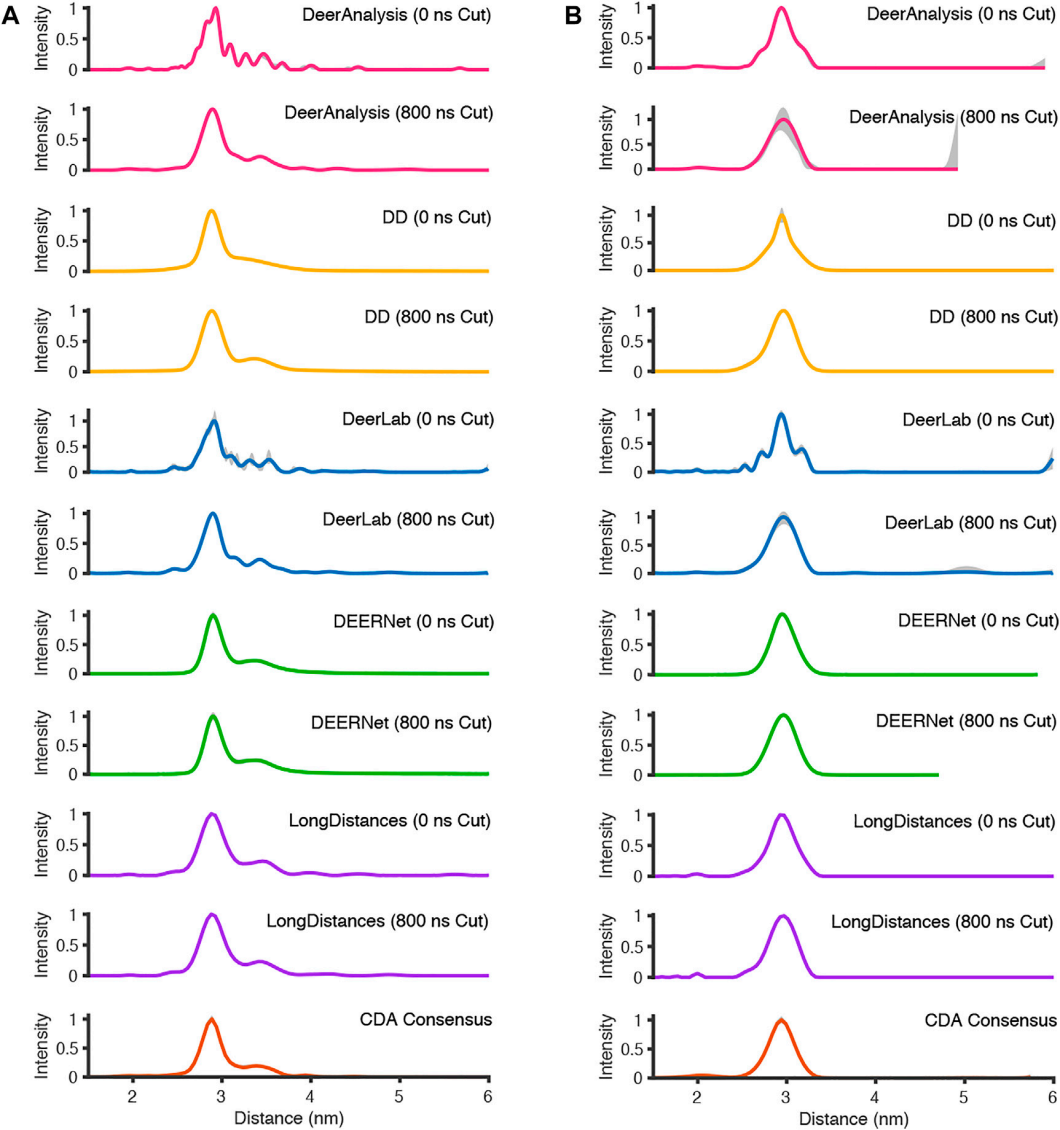
The analysis of the experimental DEER time traces using the various analysis packages (CDA Consensus is the consensus result from ComparativeDeerAnalyzer) **(A)** AGAO; **(B)** DNA.

The analysis procedures were run twice for most packages: once for the full-length experimental time traces, and once for the data with 800 ns removed from the end before analysis to remove the data points that may have been distorted by measurement artefacts. The exception to this approach was for ComparativeDeerAnalyzer which was run without any user input, and determined that 11% of the AGAO and 1% of the DNA data was cut from the end of the traces. The first test system, from the spin-labelled protein AGAO, was analyzed by all packages to have an asymmetric distance distribution (Figure 8A). The second system, spin-labelled DNA, consistently gave a more symmetric single-distance distribution (Figure 8B).

For the analysis approaches that used Tikhonov regularization (DeerAnalysis and DeerLab), where the full-length and the cut data were processed, the distance distributions became more smooth upon time trace truncation. This was also true for DD processing of the DNA data. The other analysis procedure results appeared more consistent across the AGAO and DNA data whether the data were truncated or not. DEERNet probably influenced the stability of the consensus result shown from ComparativeDeerAnalyzer. Most of the results have only small error or uncertainty levels as calculated by the programs, though DeerAnalysis for the 800 ns truncated DNA data set had a little more visible uncertainty at the long distance end.

Some statistical results are presented in Tables 6 and 7 for the AGAO and DNA results respectively. In the tables the “Overall Mean” refers to the mean of the entire distribution, where as the mean or FWHM of the main peak is the result for the most prominent distance peak in the distribution.

**TABLE 6:** Results from each program for the AGAO data, with both 0 ns cut and 800 ns cut.

	Data truncated	Overall mean (nm)	Mean main peak (nm)	FWHM main peak (nm)
DeerAnalysis	0 ns	3.06	2.90	0.19
	800 ns	3.05	2.87	0.20
DD	0 ns	3.07	2.85	0.19
	800 ns	3.07	2.85	0.24
DeerLab	0 ns	3.08	2.86	0.15
	800 ns	3.05	2.88	0.20
DEERNet	0 ns	3.15	2.91	0.19
	800 ns	3.19	2.93	0.20
LongDistances	0 ns	3.06	2.90	0.27
	800 ns	3.05	2.90	0.27
CDA Consensus	11%	3.00	2.87	0.20
Mean of Results		3.08	2.88	0.21
Standard Deviation		0.05	0.03	0.04

**TABLE 7:** Results from each program for the DNA data set, with both 0 ns cut and 800 ns cut.

	Data truncated	Overall mean (nm)	Mean main peak (nm)	FWHM main peak (nm)
DeerAnalysis	0 ns	2.93	2.95	0.27
	800 ns	2.93	2.95	0.37
DD	0 ns	2.94	2.94	0.24
	800 ns	2.93	2.93	0.34
DeerLab	0 ns	3.05	2.95	0.14
	800 ns	3.00	2.94	0.36
DEERNet	0 ns	2.97	2.96	0.32
	800 ns	2.95	2.95	0.36
LongDistances	0 ns	2.92	2.94	0.34
	800 ns	2.92	2.94	0.37
CDA Consensus	1%	2.90	2.93	0.33
Mean of Results		2.95	2.94	0.31
Standard Deviation		0.04	0.01	0.07

The standard deviation from the mean of all the AGAO and DNA results from the different programs was less than 0.1 nm for the overall mean, mean of the main peak, and FWHM of the main peak. This indicates that all the programs are performing well, or at least are in broad agreement, as they return the main aspects of the distance distribution for the AGAO and DNA, regardless of the appearance of the distributions in Figure 8. The main peak of the AGAO result was at a mean position of 2.88 nm with a standard deviation of 0.03 nm across all results/analysis methods tested. Only one result (from DEERNet) fell slightly outside one standard deviation of the mean. The mean for the overall distribution was larger than for the main peak since the AGAO distance distribution was asymmetric, as determined by all the programs. The FWHM of this peak was 0.21 nm with a standard deviation of 0.04 nm with LongDistances slightly overestimating the width, and one of the DeerLab datasets being a little more narrow. The overall mean and the mean of the main peak in the DNA result were very close together since the distribution from the DNA appeared to be more symmetric than the AGAO result. The mean of the main peak was at 2.94 nm with a standard deviation of just 0.01 nm. The FWHM for the main peak was determined to be 0.31 nm with a standard deviation of 0.07 nm. On inspection of the data in Figure 8B and the tabulated results, the variation chiefly came from the results from DD and DeerLab for the non-truncated data where the end artefacts were present.

## 4 Discussion

We have demonstrated the use of six free-to-access packages for analyzing typical DEER time traces (pairwise spin-half dipolar interactions without orientation selection) to extract distance distributions. We have shown that all six are able to extract distances and provide uncertainty bounds of some kind.

Let us first consider accessibility. DeerAnalysis, DD and DEERNet require Matlab, and DEERNet requires several additional toolboxes. LongDistances and ComparativeDeerAnalyzer’s Windows executable version are both standalone packages and so are free. DeerLab uses Python which is freely available. However, the wide range of uses of DeerLab, and the programming environment rather than a GUI, may also make it more intimidating for users without any prior experience of Python or DEER data analysis. DeerNet also does not have a GUI but offers almost no user input and so the user only needs to follow some basic protocols. DeerAnalysis, DD, ComparativeDeerAnalyzer and LongDistances all have a graphical front end and are intuitive to use once the provided guidance has been read. We hope our guide on getting started will be a useful addition to the authors’ own documentation and we feel it pertinent to emphasize that intuitive use does not necessarily mean best, or even correct, results.

To expand on the discussion of the authors’ own documentation, we consider three areas: length, difficulty, and quality. DD has the shortest documentation of the packages, though it is easy to follow and clearly lays out the processes that should be undertaken by the user. DEERNet’s documentation is short but clear and tells the user everything they need to know to do their own analyses. Further, the mathematics and neural network descriptions and explanations are neatly laid out in the associated publications. DeerAnalysis has an excellent manual included in its download zip file. The length is such that it could be read in full by the user with ease, and it plainly lays out every detail of the package. Likewise, the zip file contains a short and simple manual for the running of ComparativeDeerAnalyzer. DeerLab’s documentation is extensive yet easy to follow and is split into digestible pages and sections. The documentation for LongDistances is split into sections for each of the GUI tabs where the functional processes are described succinctly and can additionally be accessed by pressing the “Help” button. In all cases direct communication with the authors when there are questions, is possible and even encouraged. DD, DeerAnalysis and ComparativeDeerAnalyzer documentation is provided in the download file, while the documentation for the other packages are web-based and linked to from the download pages.

While DeerAnalysis and DEERNet allow for editing of the final figure outputs of the results, the other packages do not. Therefore, for future presentation of the results, it is necessary for the results to be saved as text-type files, or similar, to facilitate this. DeerNet is the only program that does not allow for the results to be saved as text-type files for further figure preparation but the .mat saved file output can be saved as text files by the user. The saved files include meta-data such as modulation depths, with the exception of DeerLab, for which the user should note down the modulation depth. In our experience the programs all presented very similar values of the modulation depth parameter for a given data set, see Tables 1–3.

The next point to consider is the user input required to run the packages. All packages allow for truncation of the data set. We did not run ComparativeDeerAnalysis in this way so as to leave that as a fully automatic process for our types of data. Truncating the artefactual experimental time traces was seen to alter the final results in our tests for the programs tested, except for DEERNet (Figure 8).

With the exception of DEERNet, which has no parameters to adjust, the packages offer the user the ability to define most of the variables, but following a workflow as defined by the user manuals and avoiding unnecessary variable changes should remove user-induced errors. Methods should be published with the data analysis results to aid reproducible and transparent data handling.

The programs, apart from DEERNet, allow for some variation in the background model with DeerLab, DeerAnalysis, and DD offering the most flexibility. Some preliminary checks with a simulated data set similar to the noise-free set used here but with a “2D” stretched exponential background (detailed in the Materials and Methods section) suggested that for small deviations from the homogeneous background the induced error was minor though the certainty bounds on the distance results improved when the correct background function was applied. The extent to which the packages are able to deal with different background forms has not been investigated more thoroughly in this work since there are a wide range of possible backgrounds, and the user is reminded to be cautious when analyzing non-standard DEER data in whatever form that takes. We note that the RIDME (relaxation induced dipolar modulation enhancement) experiment, which like DEER measures dipolar coupling frequency, suffers from a, to date, not well predicted background function and that DeerNet (and ComparativeDeerAnalyzer) has recently been expanded to include RIDMENets (Milikisyants et al., 2009; Keller et al., 2019; Ritsch et al., 2019; Keeley et al., 2022).

Now the discussion moves to the output results from the packages. First of all, we will consider the best fit results, not the uncertainty/error ranges. All the packages gave a good approximation of the distance distributions for the test data (simulated and experimental) used in this paper (see Tables 4–7). We found that by analyzing the data using multiple approaches the user is able to gain a better appreciation of the system being measured and assurance over the shape of distributions. For example, the body of results in Figure 8 clearly indicates that the AGAO distance distribution is asymmetric and the spin-labelled DNA distribution is essentially Gaussian.

DeerAnalysis and DeerLab utilized Tikhonov regularization to stabilize the ill-posed inverse transformation of the time traces to distance distributions. They use different methods for optimizing the regularization parameter, which will ultimately affect the smoothness and broadness of the distribution. In the analysis of the simulated data (Figure 7), both programs are less robust when there is noise on the data though the overall distributions remain smooth. For the experimentally-collected DEER data, the approaches tend to give over-defined results until the data are truncated to remove end-artefacts, and then the smoothness of the presumed real underlying distribution is recovered. LongDistances’ model-free fitting, which is similar to, but not the same as, Tikhonov regularization gives simulated data results that are smooth (as defined by the user) but are affected by the inclusion of noise in the data, much like DeerAnalysis and DeerLab. For the experimentally-collected results, the distributions remain relatively consistent, and appear largely unaffected by the 2 + 1 effect artefacts in the full-length result.

The parameterized-model approach of DD worked well for the data sets presented here with some improvement made when the spin-labelled DNA data was truncated. DEERNet gave consistent outputs for all the data sets. The results from DEERNet appear to stabilize the consensus result in ComparativeDeerAnalyzer.

While all the programs offer an approach to calculating uncertainty, we have seen in our tests that some of these are more useful than others. We found that running validation in DeerAnalysis was very prone to user input and quite slow compared to the other methods. The uncertainty in LongDistances often appeared to be underestimated with the methods we used, with all distance distributions given no, or very little, uncertainty. Meanwhile, the errors reported by DeerLab seem reasonable for the simulated and experimental data. DEERNet appears unaffected by the longer-time artefact, with its results remaining consistent (and presumably accurate) for the tests. The confidence intervals from DEERNet are negligible for the data sets used here.

The consensus result from ComparativeDeerAnalyzer is the result of using output from DEERNet to inform DeerLab. Only if DEERNet does not find a result will the default parameters for DeerLab follow those seen in the DeerLab documentation. This means that in general, the output from DeerLab in ComparativeDeerAnalyzer’s report will not be the same as DeerLab calculations carried out in accordance with the documentation.

While each package has its disadvantages and downfalls, they each also have advantages and strengths. DeerAnalysis, in user-defined mode, gives the user both freedom and the ability to be constrained to automatically determined parameters, but falters due to over-complicated error analysis and the potential for user bias. The advantages of DD are that it has a simple and intuitive layout, and its focus on a single analysis method limits sources of confusion for new users. The user must decide how many Gaussians to include, which is a simple process that only requires the user to test different numbers of Gaussians to determine which achieves the lowest BIC value. For the data presented in this paper, DD worked well, though some user input to truncate data containing artefacts improved the final distance distributions. LongDistances in particular provided a friendly environment for understanding the role of the smoothness parameter on the appearance of the result with a simple slider bar and mouse-based control, possibly making it a good method for teaching students the effect of parameters on DEER data analysis. DeerLab has seemingly limitless uses and capabilities (see the documentation), but it may appear off-putting to non-coders and to those new to DEER data analysis. DEERNet, while being code-based in its Spinach-form, is less intimidating and produced consistently accurate results with what appeared to be reasonable error estimates for our data sets. The downsides of DEERNet are that its nets are intrinsically somewhat blackbox, and in its full form it requires a number of Matlab toolboxes which may make its use prohibitive to some users. Incorporating parts of the last two packages and providing a “Consensus” result is ComparativeDeerAnalyzer. With the community-led white paper guiding users towards single-step analysis and multi-platform comparisons (Schiemann et al., 2021), future users may lean towards ComparativeDeerAnalyzer as a first port-of-call. We have however shown that the use of other packages may provide further insight into the data, and simpler approaches can lead to a better understanding of the final results. Also, the requirement to alter fitting parameters may be required in certain circumstances. It is not the purpose of this work to say any package is “better” than another, but rather to demonstrate how each package compares to the others, and how to get started in each.

## Data Availability

Publicly available datasets were analyzed in this study. This data can be found here: https://doi.org/10.17630/99a46d98-c92d-439a-9462-31b660dcd952.
